# Melatonin induces cell cycle arrest and suppresses tumor invasion in urinary bladder urothelial carcinoma

**DOI:** 10.18632/aging.204673

**Published:** 2023-04-21

**Authors:** Tzuo-Yi Hsieh, Wen-Wei Sung, Ya-Chuan Chang, Chia-Ying Yu, Li-Yu Lu, Chen Dong, Tsung-Hsien Lee, Sung-Lang Chen

**Affiliations:** 1 Institute of Medicine, Chung Shan Medical University, Taichung, Taiwan; 2 School of Medicine, Chung Shan Medical University, Taichung, Taiwan; 3 Department of Urology, Chung Shan Medical University Hospital, Taichung, Taiwan; 4 Department of Obstetrics and Gynecology, Chung Shan Medical University, Taichung, Taiwan; 5 Division of Infertility Clinic, Lee Women’s Hospital, Taichung, Taiwan

**Keywords:** melatonin, bladder cancer, apoptosis, NF-κB, HIF-1α

## Abstract

Urinary bladder urothelial carcinoma (UBUC) encompasses about 90% of all bladder cancer cases, and the mainstream treatment is the transurethral resection of the bladder tumor followed by intravesical instillation. High rates of mortality, recurrence, and progression in bladder cancer have stimulated the search for alternative adjuvant therapies. The aim of this study was to investigate the potential of melatonin as adjuvant therapy in bladder cancer. Cell viability and clonogenic ability were assessed by an MTT assay and colony formation. Cell cycle and apoptosis analysis were performed by flow cytometry and Hoechst 33342 staining, while cell metastasis capacity was measured by wound healing and transwell assays. Potential mechanisms were investigated by an oncology array and verified via western blotting. The melatonin treatment significantly reduced T24 and UMUC3 bladder cancer cell proliferation and clonogenic ability. G1 arrest and sub-G1 accumulation in the T24 and UMUC3 cells led to cell proliferation suppression and cell death, and Hoechst 33342 staining further verified the apoptosis induction directly by melatonin. Moreover, melatonin weakened cell motility and invasiveness. Based on the oncology array results, we demonstrated that melatonin exerts its anti-cancer effect by down-regulating the HIF-1α and NF-κB pathways and downstream pathways, including Bcl-2, leading to cell cycle arrest and apoptosis induction in the UBUC cells. Overall, these findings support the potential of melatonin as adjuvant therapy in bladder cancer.

## INTRODUCTION

According to 2020 GLOBOCAN data, bladder cancer is the 10th most common cancer and the 14th most deadly with an estimated 573,278 diagnoses and 212,536 deaths [1]. The worldwide age-standardized incidence rates and mortality rates (per 100,000 persons/year) are 9.5 and 3.3 for men and 2.4 and 0.9 for women, respectively [1]. According to the SEER (Surveillance, Epidemiology, and End Results) database, the 5-year overall survival rate between 2011–2017 in the United States was 77.1% and the 10-year overall survival rate was barely 70% [2]. Among bladder cancer diagnoses, urinary bladder urothelial carcinoma (UBUC) accounts for more than 90% and squamous cell carcinoma accounts for 5% [3]. Current treatments include smoking cessation, complete transurethral resection, single immediate instillation of chemotherapy, and intravesical Bacille Calmette-Guerin [4]. According to the European Organization for Research and Treatment of Cancer and the Genito-Urinary Cancer Group model, the rate of recurrence and progression at 5 years can range from 31% to 78% and 0.8% to 45%, respectively, depending on the severity of the disease [5]. Due to the high rates of recurrence and progression, adjunct therapy is needed to improve the prognosis for this disease.

Melatonin is an indole hormone synthesized and secreted by the human pineal gland in response to darkness [6]. The suprachiasmatic nucleus of the hypothalamus regulates the synthesis and secretion of melatonin according to circadian rhythms [7]. Melatonin acts as a cell protector for healthy cells through anti-oxidative, hematopoietic, antigenotoxic, and immunomodulatory effects that influence longevity. Multiple studies have shown the anticancer effects of melatonin in multiple cancers, including breast, prostate, liver, lung, and colorectal cancers [8]. In malignant cells, melatonin has pro-apoptotic effects through it terminating the ROS-induced Akt signaling pathway, which reduces the expression of anti-apoptotic proteins, such as Bcl2, PCNA, and cyclin D1, and elevates pro-apoptotic proteins, including Bax [9]. In addition, melatonin induces apoptosis in malignant cells through the regulation of the NF-κB, COX-2/PGE2, Apaf-1/caspase-9, and PI3K/AKT/mTOR pathways [10, 11]. Moreover, melatonin impairs chemotherapy resistance in advanced nasopharyngeal carcinoma cells by suppressing the Wnt/β-catenin pathway [12]. However, while many clinical trials have investigated the benefits of melatonin in the treatment of various cancers, the underlying mechanisms of melatonin in bladder cancer are mainly unknown.

High rates of mortality, recurrence, and progression in bladder cancer have promoted the search for alternative adjuvant therapies that can increase the efficacy of bladder cancer treatments. The main purpose of the current study was to investigate the anti-cancer effects of melatonin in human bladder cancer cells, including T24 and UMUC3, along with evaluating its effects on cell cycle phase distribution, apoptosis, and metastatic ability. Furthermore, we applied a proteome profiler oncology array to clarify the underlying molecular mechanism regulated by melatonin in the UBUC cells. These results helped us to elucidate the anti-tumor role of melatonin in bladder cancer and provide a new target for alternative adjuvant therapy.

## METHODS

### Cell culture

UBUC cell lines T24 and UMUC3 were purchased from the American Type Culture Collection (ATCC, VA, USA). They were cultured and stored in line with the supplier’s instructions. The T24 and UMUC3 cells were grown in RPMI-1640 medium, and 10% fetal bovine serum (FBS), 100 μg/ml streptomycin, 0.1 mM NEAA, 100 U/ml penicillin, 1 mM sodium pyruvate, and 2 g/ml NaHCO_3_ (Thermo Fisher Scientific Inc., Waltham, MA, USA) were added to the medium.

### MTT assay

An MTT assay was performed to evaluate the cytotoxicity of melatonin on the T24 and UMUC3 cells by assessing cell viability. Briefly, we used 96-well plates to seed 1 × 10^4^ cells overnight and exposed them to melatonin (0, 0.5, 1, and 2 mM, respectively) for 24 hours separately. MTT solution (0.5 mg/mL) was then added and incubated for 3 hours at 37°C in an incubator. The reaction was stopped by the removal of the supernatant, which was followed by the addition of DMSO to dissolve all of the purple crystals. The absorbances of the cells at a wavelength of 570 nm were measured by a microplate reader to determine cell viability. All of the experiments were performed in triplicate.

### Colony formation assay

A colony formation assay was performed to detect the proliferation ability of the cells. Bladder cancer cells were seeded in a six-well plate at 250 cells/well and 500 cells/well of T24 and UMUC3 cells, respectively. After being treated with melatonin (0, 0.5, 1, and 2 mM) for 10 days, the cells were washed with phosphate buffered saline (PBS) twice. Iced ethanol was then added (95%) for 20 minutes, and the cells were stained with 0.5% crystal violet for 10 minutes. All the experiments were performed in triplicate, and counts of the colonies were used to assess the anti-proliferation effect of melatonin on the UBUC cell lines.

### Flow cytometry analysis

To determine the distribution of the cells in their cycle phases, a flow cytometry analysis was performed using a flow cytometer (FACSCantoTMII Cell Analyzer, BD Biosciences, Franklin Lakes, NJ, USA). The T24 and UMUC3 cells were treated with melatonin for 24 or 48 hours. The treated cells were collected and fixed with ice-cold 70% ethanol and then stained with propidium iodide (PI) staining buffer (0.5 mg/ml DNase-free RNase A and 0.4 μg/ml PI in PBS). The distribution of the cell cycle phases was detected by a flow cytometer afterward. The charts were depicted by FlowJo software (BD Biosciences, Franklin Lakes, NJ, USA). All of the experiments were performed in triplicate.

### Hoechst 33342 staining

Hoechst 33342 staining was used to detect the morphological changes in cells’ nuclei. The T24 and UMUC3 cells were seeded in six-well plates in a concentration of 2.5 × 10^5^ cells/well and treated with melatonin 1 mM or 2 mM for 48 hours. After being washed with PBS, the cells were stained with Hoechst 33342 (10 μg/ml) and incubated for 20 minutes in an incubator. Nuclear condensation and a reduction in nuclear volume were observed in the apoptotic cells, whereas the normal cells showed ordinary nuclear and uniform fluorescence under fluorescence microscopy (ImageXpress PICO, San Jose, CA, USA) at excitation wavelengths of 350–390 nm and emission wavelengths of 420–480 nm [13]. The pictures from five random fields were observed to assess the apoptotic rates of the T24 and UMUC3 cells.

### Western blotting

The melatonin-treated T24 and UMUC3 cells were lysed in 200 μl of RIPA buffer that contained a protease inhibitor cocktail and phosphatase inhibitor (Roche Applied Science, Mannheim, Germany). We scraped the lysed cells into an Eppendorf flask and then centrifuged it at 10,400 rpm for 20 minutes at 4°C. The supernatant was saved and made into samples containing 15 μg of protein, which were separated by 6–15% SDS-PAGE and then electroblotted onto polyvinylidene fluoride membranes (Merck Millipore, Burlington, MA, USA). The blocking of the membranes was done by submerging them into 5% non-fat dry milk for 2 hours at room temperature. The membranes were then incubated with the following primary antibodies (diluted 1:1000): cyclin E (A14225, ABclonal), CDK2 (A18000, ABclonal), p21 (10355-1-AP, Proteintech), p53 (tcea17012, Taiclone), IκBα (A11397, ABclonal), p-IκBα (AP0999, ABclonal), p50 (A6667, ABclonal), p-p50 (AP0125, ABclonal), p65 (A19653, ABclonal), p-p65 (AP0215. ABclonal), HIF1α (3046910, BD Biosciences), MCL-1 (16225-1-AP, Proteintech), Bcl-2 (12789-1-AP, Proteintech), Bcl-XL (A0209, ABclonal), Bax (A19684, ABclonal), claspin (A17202, ABclonal), survivin (A1551, ABclonal), N-cadherin (22019-1-AP, Proteintech), vimentin (10366-1-AP, Proteintech), slug (#9585, Cell Signaling), and ZEB1 (#70512, Cell Signaling) overnight at 4°C. They were incubated the next day with Goat anti-Mouse (C04001, Croyez) and Goat anti-Rabbit (C04003, Croyez) secondary antibodies at room temperature for 1 hour first and then followed with Immobilon Western Chemiluminescent HRP Substrate (Merck Millipore, Burlington, MA, USA). ImageQuant LAS4000 was used to quantify the results (GE Healthcare, Marlborough, MA, USA).

### Proteome profiler human XL oncology array for proteome profiling

A Proteome Profiler Human XL Oncology array (R&D Systems, Minneapolis, MN, USA) was used to measure the expression of cancer-related proteins. The relative expression levels of 84 human cancer-related proteins can be detected in duplicate by the antibodies on the array’s nitrocellulose membrane. The T24 and UMUC3 cell lysates that had been treated with melatonin for 48 hours were collected for analysis after harvesting, and 200 μg of total protein from the cells was immersed with the Proteome Profiler Human XL Oncology array. We washed away the unbound antibodies and followed this by incubation of the membranes in horseradish peroxidase-conjugated secondary antibody. Following the addition of a chemiluminescent reagent, the cells were examined with an ImageQuant LAS4000 instrument (GE Healthcare, Marlborough, MA, USA). ImageJ software (National Institutes of Health, Bethesda, MD, USA) was used to analyze the integrated density of each membrane.

### Wound healing assay

Wound healing tests were used to examine the UMUC3 and T24 cell migrations. We used ibidi culture inserts (Ibidi, Gräfelfing, Germany) for seeding the T24 and UMUC3 cells at a density of 30,000 cells per well. The inserts were removed after 24 hours of culturing, and the T24 and UMUC3 cells were treated with 0.5, 1, and, 2 mM melatonin in 0.5% FBS medium. We captured images of the wounds with a microscope at 0, 24, and 48 hours after melatonin treatment, which were later analyzed with ImageJ software version 1.52a (National Institutes of Health, Bethesda, MD, USA). T24 was calculated according to migration area while UMUC3 was calculated according to cell number. We conducted each experiment in triplicate to confirm the precision of the study results.

### Transwell migration/invasion assay

Cell migration/invasion was analyzed by transwell assays (Cat# 3422, Corning, NY, USA). The cells were suspended in serum-free medium containing melatonin (0, 0.5, 1, and 2 μM). For the migration assay, 100 μl of cells were plated in the upper chamber (2.5~5 × 10^4^ cell/well), while the lower chamber was loaded with 600 μl of 10% FBS as chemoattractant, and the cells were allowed to migrate for 16 hours. For the invasion assay, the upper side of the filter was covered with 0.2% Matrigel (Collaborative Research, MA, USA) diluted in FBS containing culture medium, and the cells were allowed to invade for 24 hours. The cells that adhered to the underside of the transwell membrane were fixed with 95% ethanol and stained with 1% crystal violet solution, and the cells on the upper side of the membrane were removed by wet cotton swabs. Each experiment was performed in triplicate, and the number of migration cells/mm^2^ on whole transwell surface for each group was tabulated by TissueFAXS Plus software (Vienna, Austria).

### Statistical analysis

All data were presented as the mean ± SD for the triplicate tests, and the statistical analyses were performed using IBM SPSS software version 20.0 (Armonk, NY, USA). A student’s *t*-test was used for the data analysis. All tests were two-sided, and a *p* value < 0.05 was considered statistically significant (^*^p < 0.05; ^**^p < 0.01; ^***^p < 0.001).

### Data availability statement

All data analyzed are included in this article, and additional information is available upon request.

## RESULTS

### Melatonin prohibits cell progression of UBUC cells *in vitro*

**Figure 1:**
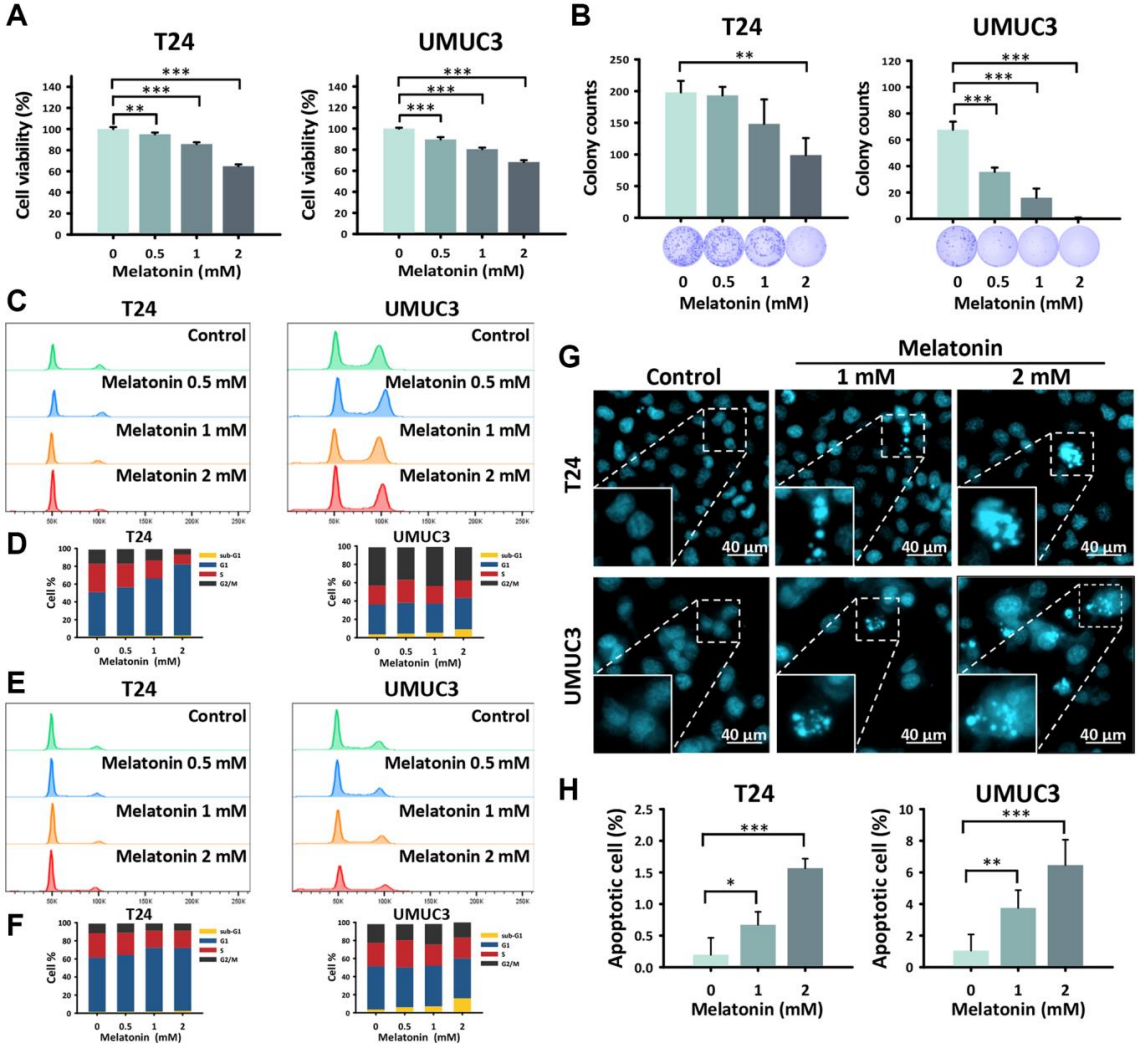
**Melatonin prohibited cell proliferation and promoted apoptosis in UBUC cells.** (**A**) An MTT assay was performed to detect the cell viability of the T24 and UMUC3 cells treated with melatonin at 0, 0.5, 1, and 2 mM for 24 hours. (**B**) The T24 and UMUC3 cells were exposed to 0, 0.5, 1, and 2 mM melatonin for 10 days and were quantitatively analyzed by a colony formation assay. The cell cycle distribution of (**C**, **D**) 24-hour and (**E**, **F**) 48-hour melatonin-treated UBUC cell lines was assessed by flow cytometry. (**G**) Hoechst 33342 staining was used to detect apoptotic cells in the melatonin-treated UBUC cells, and (**H**) the apoptotic rate of each group is shown. Bars represent as mean ± SD. ^*^p < 0.05, ^**^p < 0.01, ^***^p < 0.001.

Previous studies have shown that melatonin has a potential protective effect in colorectal cancer and breast cancer [14–16]. Herein, we aimed to evaluate the anti-tumor effect of melatonin and clarify the underlying mechanisms of melatonin in UBUC cells. First, we chose T24 and UMUC3 cells as our UBUC cell lines to evaluate the anti-cell growth and anti-cell proliferation effects of melatonin. As assessed by an MTT assay, the cell viability of the T24 and UMUC3 cells declined significantly with an increase in dosage after a 24-hour melatonin treatment, which is shown in Figure 1A (64.8% and 68.3%, *p* < 0.001 and *p* < 0.001, respectively). As shown in Figure 1B, the clonogenic ability of the T24 and UMUC3 cells was also suppressed after being treated with melatonin for 10 days, which was demonstrated by the significantly decreased colony counts in the group treated with 2 mM melatonin (99.0 and 0.3, *p* < 0.01 and *p* < 0.001, respectively) as compared to the control groups (198.0 and 67.7, respectively).

### Melatonin promotes apoptosis in T24 and UMUC3 cells

To better explore the cytotoxicity of melatonin, we proceeded to examine the cell cycle distribution of the UBUC cells using flow cytometry to clarify the cause of the cell progression suppression. The results revealed that 24-hour (Figure 1C, 1D) and 48-hour (Figure 1E, 1F) melatonin treatments increased G1 phase and sub-G1 phase accumulation in the T24 and UMUC3 cells, which was indicative of cell cycle arrest and cell death, respectively. The G1 phase significantly increased in the T24 cells after the 2 mM melatonin treatments of 24 hours and 48 hours (79.9 % and 69.6 %, *p* < 0.001 and *p* < 0.01, respectively) as compared to the control groups (49.6% and 59.9%, respectively). The sub-G1 phase increased significantly in a time-dependent manner in the UMUC3 cells after a 2 mM melatonin treatment for 24 hours and 48 hours (9.2% and 16.0%, *p* < 0.001 and *p* < 0.001, respectively) as compared to the control groups (3.6% and 3.5%, respectively). Furthermore, Hoechst 33342 staining confirmed that melatonin induced cell death by promoting cell apoptosis accompanied by morphological changes in the nuclei, including chromatin condensation and nuclear volume reduction in the UBUC cells (Figure 1G, 1H). The results indicate that the apoptotic cells in the T24 and UMUC3 lines increased significantly in the group treated with 2 mM for 48 hours (1.6 %, *p* < 0.001 and 6.5 %, *p* < 0.001, respectively) as compared to the control groups (0.2% and 1.0%, respectively).

### Melatonin mitigates metastatic ability in UBUC cells

**Figure 2:**
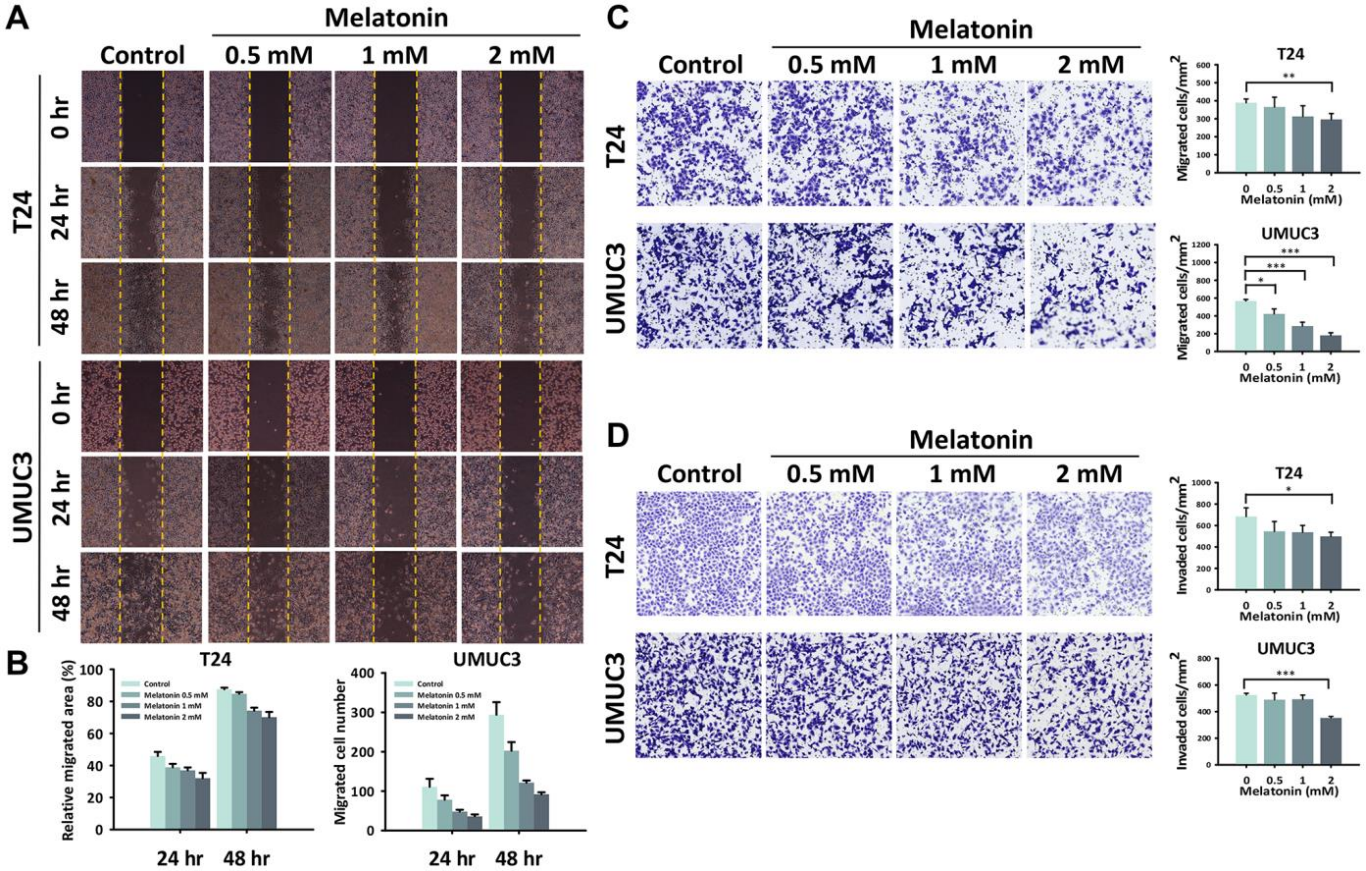
**Melatonin weakened the cell migration/invasion abilities of the UBUC cells.** (**A**) The migration ability altered by melatonin in the UBUC cells was detected by a wound healing assay, and (**B**) the relative migrated area and migrated cell numbers were quantified at 24 and 48 hours. A transwell assay was used to evaluate the (**C**) migration and (**D**) invasion abilities of the UBUC cells with a melatonin treatment. The migrated and invaded cells that crossed the membrane were quantified and are depicted as bar plots. The bars represent mean ± SD. ^*^p < 0.05, ^**^p < 0.01, ^***^p < 0.001.

Considering that the metastatic ability of a cell plays an important role in tumor malignancy and T24 and UMUC3 cells are highly metastatic cells, we wondered whether melatonin had any effect on migration and invasion capacity. Hence, we performed wound healing and transwell migration/invasion assays to evaluate the anti-metastatic effects of melatonin in the UBUC cells. As assessed by the wound healing assay, we found that melatonin inhibited the cell migration ability of the UBUC cells in a time- and dose-dependent manner (Figure 2A). Quantitative analysis (Figure 2B) showed that 2 mM melatonin significantly decreased the area of T24 migrated cells and the number of UMUC3 migrated cells (*p* < 0.001 and *p* < 0.001, respectively) as compared to the control groups at both 24- and 48-hour time points. The anti-migration effect of melatonin was additionally confirmed by the transwell migration assay (Figure 2C). A significant decrease in the migrated cell number per mm^2^ was observed in the T24 and UMUC3 cells within 24 hours of a 2 mM melatonin treatment (389.2 and 567.7, *p* < 0.01 and *p* < 0.001, respectively) as compared to the control groups (296.3 and 180.2, respectively). Furthermore, we applied the transwell invasion assay to measure the capacity for cell motility and invasiveness toward a chemo-attractant gradient in order to mimic the *in vivo* invasive behavior of cancer cells invading local tissues by degrading the ECM protein component (Figure 2D). After quantification, the number of T24 and UMUC3 cells treated with 2 mM melatonin for 24 hours that had transversed to the basement membrane was significantly decreased (683.6 and 525.8, *p* < 0.05 and *p* < 0.001, respectively) as compared to controls (498.3 and 352.4, respectively.

### Identification of cancer-related proteins altered by melatonin treatment

**Figure 3:**
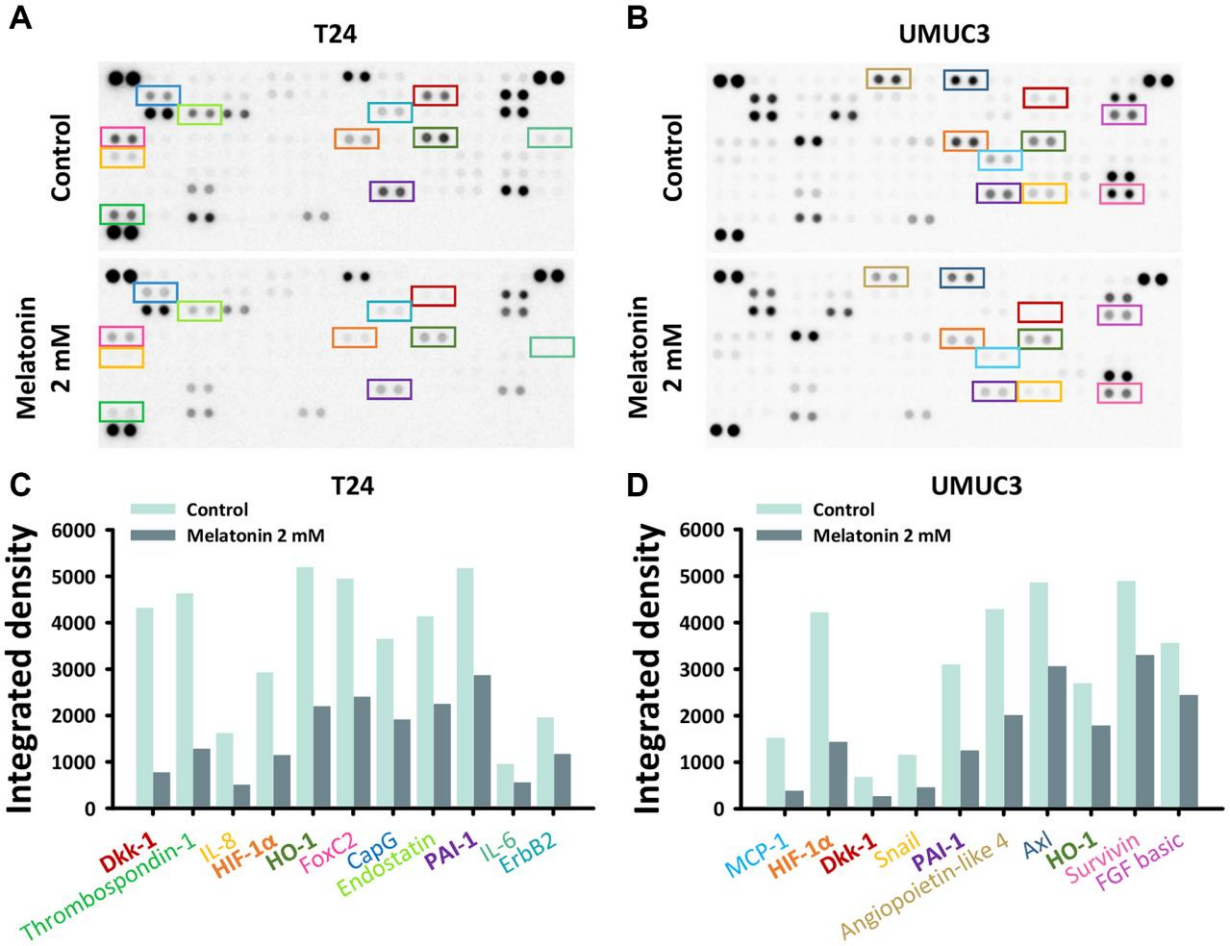
**The oncology-related protein expressions altered by melatonin were measured by a proteome profiler oncology array.** The relative expression of 84 human cancer-related proteins regulated by a 48-hour melatonin treatment (0 or 2 mM) was determined for (**A**) T24 and (**B**) UMUC3 cells. Protein expression changes in the (**C**) T24 and (**D**) UMUC3 cells were quantified and are depicted as bar plots.

With the previous confirmation of the melatonin-induced apoptotic and anti-migration/invasion effects in the UBUC cells, we afterward used the Proteome Profiler Human XL Oncology array for proteome profiling to determine the underlying molecular mechanisms regulated by the melatonin treatment. Figure 3A, 3B shows an analysis of the expression levels of 84 human cancer-related proteins that compares parental cells and the T24 and UMUC3 cells after separate 48-hour 2 mM melatonin treatments. Based on the quantified results shown in Figure 3C, 3D, the downregulation of the upstream HIF-1α can be observed in both UBUC cell lines as compared to the control groups. Hence, we hypothesized that melatonin downregulates HIF-1α expression, a well-known oncology marker, in UBUC cells to provide a pro-apoptosis role by affecting its downstream pathways, which possess anti-apoptotic effects, including the Bcl2 pathway [17]. Furthermore, we further suspected that the NF-κB pathway, which shares numerous downstream target genes with HIF-1α [18] and is involved in tumorigenesis, may be affected by melatonin treatment.

### Melatonin exerts its anti-cancer effects through cell cycle regulation and HIF-1α downstream signaling suppression

**Figure 4:**
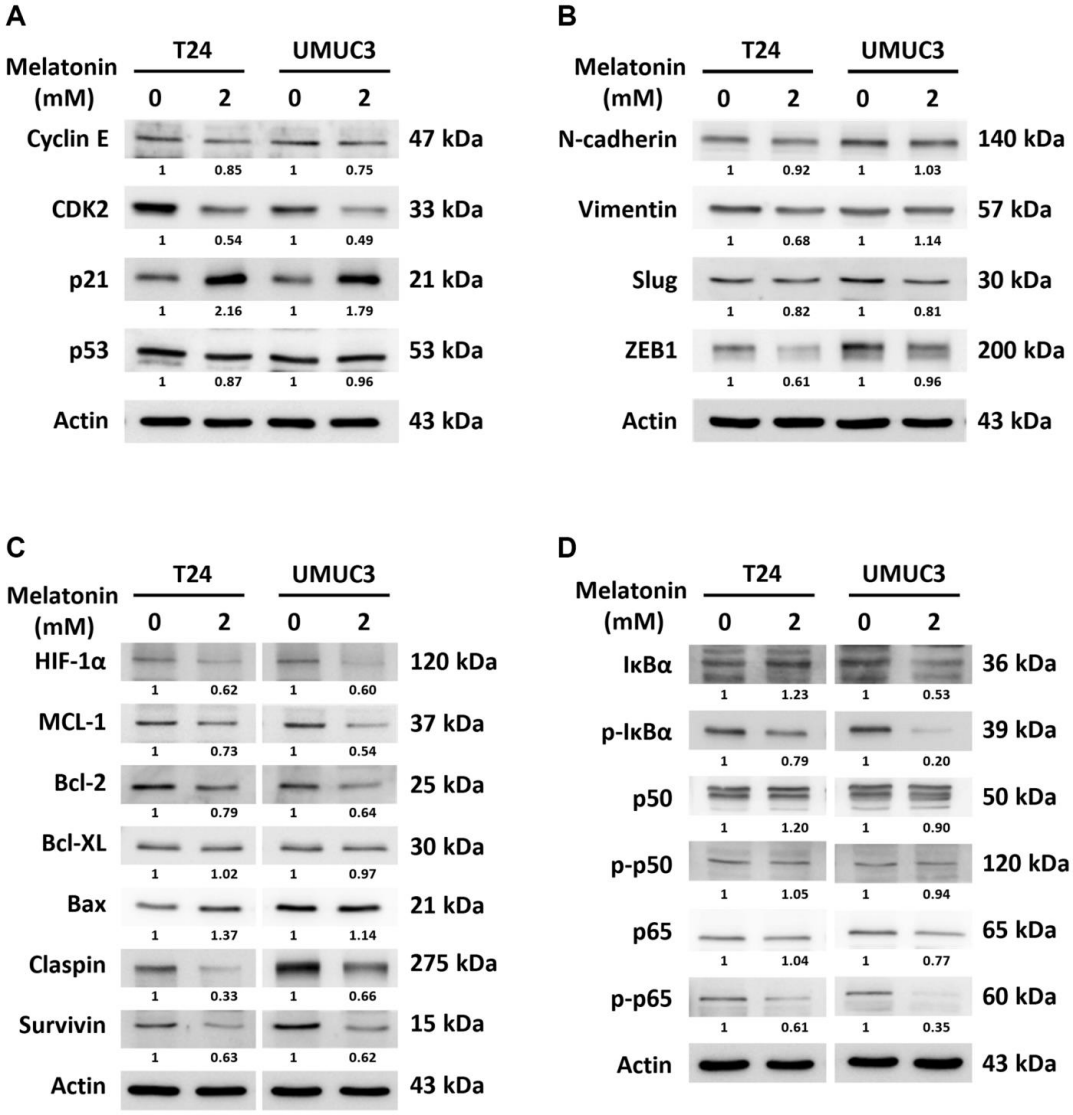
**Validation of cell cycle, apoptosis, and metastasis-related protein expressions via western blotting.** The protein expressions changed by a 24- or 48-hour melatonin treatment on UBUC cells were measured via western blotting, including of (**A**) cell cycle arrest-related proteins, (**B**) metastasis-related proteins, and (**C**, **D**) apoptosis-related proteins.

A more comprehensive verification was therefore performed via western blotting to reveal and confirm the underlying mechanisms altered by melatonin in the UBUC cell lines. With a 2 mM melatonin treatment, the protein expressions of cyclin E and CDK2, which are required for a cell transition from the G1 to S phase of the cell cycle, were significantly reduced. In addition, the protein expression of p21, which binds to and inhibits the activity of all cyclin/CDK complexes, was significantly increased, leading to the cell cycle arrest in the UBUC cells (Figure 4A). To validate the cell migration/invasion-related protein expression, we checked the epithelial-to-mesenchymal transition proteins, including N-cadherin, vimentin, slug, and ZEB1. The results indicate that slug and ZEB1, which cooperate in regulating cell-cell adhesion, were downregulated by the 24-hour melatonin treatments in both cells. However, there were no significant differences in the N-cadherin and vimentin protein expressions despite that the wound healing and transwell assays showed that the melatonin treatment significantly inhibited the cell migration ability of both cells (Figure 4B). Finally, according to the oncology array results, we measured the HIF-1α, NF-κB, and Bcl2 pathways to validate the underlying mechanisms of melatonin in the UBUC cells. As shown in Figure 4C, melatonin regulated the protein expressions of the HIF-1α and Bcl2 pathways’ downstream proteins, including MCL1, Bcl-XL, Bax, claspin, and survivin, which possess anti-cancer effects in numerous cancer cell types. Moreover, the 48-hour melatonin treatment decreased the expression level of the NF-κB signaling pathway, demonstrating its anti-cancer role in both UBUC cell lines (Figure 4D).

## DISCUSSION

The present study provides the first evidence demonstrating that melatonin reduces tumor growth through the suppression of the HIF-1α and NF-κB pathways in bladder cancer cell lines. UBUC is known for high rates of mortality, recurrence, and progression with current intravesical therapy. The outcomes of the present study demonstrate the underlying mechanisms of melatonin’s anti-proliferation effect, which is through the activation of cell cycle arrest and apoptosis by the suppression of the HIF-1α and NF-κB pathways. These optimal results support the potential for using melatonin as adjuvant therapy for UBUC.

Melatonin has been shown to have a potential therapeutic effect for various cancers in many past studies. For example, melatonin inhibited the cellular proliferation of pancreatic carcinoma cells [19] and induced apoptosis in human renal cancer Caki cells to suppress tumor proliferation through the upregulation of Bim protein expression both transcriptionally and translationally [20]. Kim et al. demonstrated that melatonin treatment combined with ER stress inducers enhanced apoptosis in B16F10 murine melanoma cells through the regulation of the PI3K/AKT/mTOR pathway [10]. Wang et al. showed that melatonin stimulated apoptosis in MDA-MB-361 breast cancer cells through the suppression of the COX-2/PGE2, p300/NF-κB, and PI3K/AKT pathways and activation of the Apaf-1/caspase-dependent pathway [11]. Cos et al. showed that melatonin inhibits the growth of human estrogen-responsive MCF-7 breast cancer through the regulation of the p53-p21 pathway [21]. Another study showed that melatonin increased the expression of p53 and p21 proteins in HepG2 human hepatocarcinoma cells to cause cell cycle arrest in the G2/M phase [22]. Related to our research on the effect of melatonin on bladder cancer, melatonin in combination with curcumin inhibited the IKKβ/NF-κB/COX-2 pathways in T24, UMUC3, and 5637 cells [23]. Another study found that melatonin inhibited the human bladder cancer cell lines HT1376, HT1197, RT4, and T24 by reducing cell proliferation, invasion, and migration through the suppression of the AKT/MMP9 pathway by decreasing ZNF746 protein expression [24]. In addition, melatonin in combination with valproic acid activated the Wnt and Raf/MEK/ERK pathways in UC3 bladder cancer cells to enhance cytotoxicity [25].

Our work suggests that melatonin reduced cell proliferation by promoting cell cycle arrest and apoptosis (Figure 1) and decreasing migration and invasion (Figure 2). These effects were verified by examining cell cycle, apoptosis, and metastasis-related protein expressions via western blotting (Figure 4A, 4B). The proposed pathway of the anticancer effects of melatonin is associated with the suppression of the HIF-1α and NF-κB pathways, which leads to the downregulation of MCL1, Bcl2, claspin, and survivin and the upregulation of Bax protein expression in UBUC cells (Figure 4C, 4D). HIF-1α regulates the excessive pro-carcinogenic genes that are involved in cell proliferation and survival, angiogenesis, invasion, metastasis, and metabolism [26, 27]. HIF-1α is often overexpressed in cancer, and the expression level is associated with poor outcomes in several types of cancer, including bladder cancer [28, 29]. A constitutive activated NF-κB signaling pathway promotes cancer cell survival in many types of cancer by leading to the activation of several anti-apoptotic genes, such as cIAP1/2, XIAP, c-FLIP, and members of the Bcl2 family [30, 31]. In fact, HIF-1α and NF-κB share numerous target genes involved in tumorigenesis [18]. The intricate crosstalk between the HIF-1α and NF-κB pathways has been demonstrated [31, 32]. For example, hypoxia-induced HIF-1α expression can be controlled by NF-κB at transcriptional levels in smooth muscle cells and embryonic kidney cells [33, 34]. An *in vivo* study using IKKβ knockout mice also confirmed that NF-κB is a transcriptional activator necessary for HIF-1α protein expression [35]. Conversely, the knockout of HIF-1α in murine neutrophils results in a reduction of NF-κB signaling pathway activity [36]. NF-κB activation was limited in HIF-1α deficient gastric cancer cells upon treatment with 5-FU [37]. Furthermore, NF-κB and HIF-1α can be each other’s upstream. NF-κB regulates HIF-1α and vice versa, making the crosstalk between these molecules more complex.

In the present study, the Proteome Profiler Human XL Oncology array was applied to more comprehensively explore the underlying molecular mechanisms. DKK1 expression in both cell lines was suppressed to varying degrees (Figure 3). According to the data from the oncology array, we suspect that other pathways, such as thrombospondin-1, CCL2/MCP-1, HO-1/HMOX1, Serpin E1/PAI-1, FoxC2, and angiopoietin-like 4, may play a role in diverting these two cell lines responses to melatonin treatment. Further experiments are needed to confirm other possible pathways that melatonin may induce in bladder cancer. DKK1 was originally considered as a tumor suppressor as its expression level is reduced in gastrointestinal tumors and frequently silenced in cancer cells. However, more evidence had shown that DKK1 may play a role in cancer progression as it inhibits the activation of β-catenin to allow cancer cells to have stem cell-like properties to avoid natural killer cells [38]. This may explain why in a previous study, muscle invasive and high-grade urothelial carcinoma patients had higher DKK1 serum levels as compared to controls. Furthermore, patients with high serum DKK1 were associated with poorer disease-free survival [39].

In addition to the anti-cancer effect of melatonin observed in this study, previous studies have demonstrated melatonin also played a role in enhancing the efficacy and reducing the adverse effects of chemotherapy [40–42]. Melatonin is cardioprotective against damages caused by anthracycline-related chemotherapy through elevation of ST segment and reduction of R-amplitude, decrement of cardiac injury markers in the serum level, enhancement of antioxidant enzyme activity, lowering of lipid peroxidation and causing changes in the serum lipid profiles in rats [43–45]. Moreover, melatonin was shown to decrease bladder oxidative stress and lower the activities of nitric oxide synthase and peroxynitrite, while increasing the heme oxygenase-1 (HO-1) expression level, which significantly reduced the bladder symptoms and histological damage caused by cyclophosphamide-induced cystitis in rats [46, 47]. Furthermore, activation of Nrf2/HO-1 pathway by melatonin was illustrated to reduce cisplatin-induced nephrotoxicity in rats [48]. Also, melatonin lowered nitrosative stress, poly (ADP-ribose)-polymerase (PARP) activation, and protein tyrosine nitration to reduce methotrexate-induced small intestinal damage in rats [49]. Besides, the pretreatment of melatonin was shown to reduce methotrexate-induced oxidative stress, regulate antioxidant enzyme activity and enhance myeloperoxidase activity, which may decrease methotrexate-induced renal damage in rats [50, 51]. Therefore, the protective effect of melatonin against the side effects of common chemotherapeutic agents in animal studies raises the potential of using melatonin as adjuvant therapy for UBUC.

One limitation of our *in vitro* study was that T24 and UMUC3 cells are not full representations of all UBUC types. An *in vitro* study also does not fully reflect the effect of melatonin in human UBUC. Moreover, we did not perform an animal model to test the efficacy of melatonin. Furthermore, we did not investigate the intermediate protein expressions in our inference of the possible pathways, and thus, our findings may be limited due to a lack of direct evidence. Even with these limitations, this study provides more evidence about the therapeutic role of melatonin in bladder cancer for further study.

## CONCLUSION

Melatonin can induce cell apoptosis and decrease the malignant potential of UBUC cells, including in cell proliferation and migration capability. These anti-cancer effects may be exerted by down-regulating the HIF-1α and NF-κB pathways and their downstream pathways. Our findings support the potential of melatonin as adjuvant therapy for bladder cancer.
